# 1,3,4-Oxadiazole and 1,3,4-Thiadiazole Nortopsentin Derivatives against Pancreatic Ductal Adenocarcinoma: Synthesis, Cytotoxic Activity, and Inhibition of CDK1

**DOI:** 10.3390/md21070412

**Published:** 2023-07-19

**Authors:** Daniela Carbone, Camilla Pecoraro, Giovanna Panzeca, Geng Xu, Margot S. F. Roeten, Stella Cascioferro, Elisa Giovannetti, Patrizia Diana, Barbara Parrino

**Affiliations:** 1 Department of Biological, Chemical and Pharmaceutical Sciences and Technologies (STEBICEF), University of Palermo, Via Archirafi 32, 90123 Palermo, Italy; camilla.pecoraro@unipa.it (C.P.); giovanna.panzeca@unipa.it (G.P.); stellamaria.cascioferro@unipa.it (S.C.); patrizia.diana@unipa.it (P.D.); barbara.parrino@unipa.it (B.P.); 2 Department of Medical Oncology, Cancer Center Amsterdam, Amsterdam UMC, VU University Medical Center (VUmc), De Boelelaan 1117, 1081 HV Amsterdam, The Netherlands; g.xu1@amsterdamumc.nl; 3 Department of Hematology, Cancer Center Amsterdam, Amsterdam UMC, VU University Medical Center (VUmc), De Boelelaan 1117, 1081 HV Amsterdam, The Netherlands; m.roeten@amsterdamumc.nl; 4 Cancer Pharmacology Laboratory, Fondazione Pisana per la Scienza, Via Ferruccio Giovannini 13, 56017 Pisa, Italy

**Keywords:** 1,3,4-oxadiazole, 1,3,4-thiadiazole, nortopsentin analogs, inhibition of migration, PDAC antiproliferative activity, inhibition of CDK1 expression

## Abstract

A new series of nortopsentin analogs, in which the central imidazole ring of the natural lead was replaced by a 1,3,4-oxadiazole or 1,3,4-thiadiazole moiety, was efficiently synthesized. The antiproliferative activity of all synthesized derivatives was evaluated against five pancreatic ductal adenocarcinoma (PDAC) cell lines, a primary culture and a gemcitabine-resistant variant. The five more potent compounds elicited EC_50_ values in the submicromolar–micromolar range, associated with a significant reduction in cell migration. Moreover, flow cytometric analysis after propidium iodide staining revealed an increase in the G2-M and a decrease in G1-phase, indicating cell cycle arrest, while a specific ELISA demonstrated the inhibition of CDK1 activity, a crucial regulator of cell cycle progression and cancer cell proliferation.

## 1. Introduction

Many new compounds used in drug therapy to treat different cancer types have been derived directly or indirectly from natural sources, modifying the molecular structure of natural compounds, or using their structures as lead compounds. Out of all the small-molecule drugs approved for various diseases in the past 30 years, only 17% are classified as purely synthetic compounds, while the remaining 83% are either natural products themselves or synthetic compounds derived from natural products [1]. In particular, marine sources have provided unique secondary metabolites with significant biological activities, and more than 60% of FDA-approved cancer medicines are derived from marine natural products [2].

**Figure 1:**
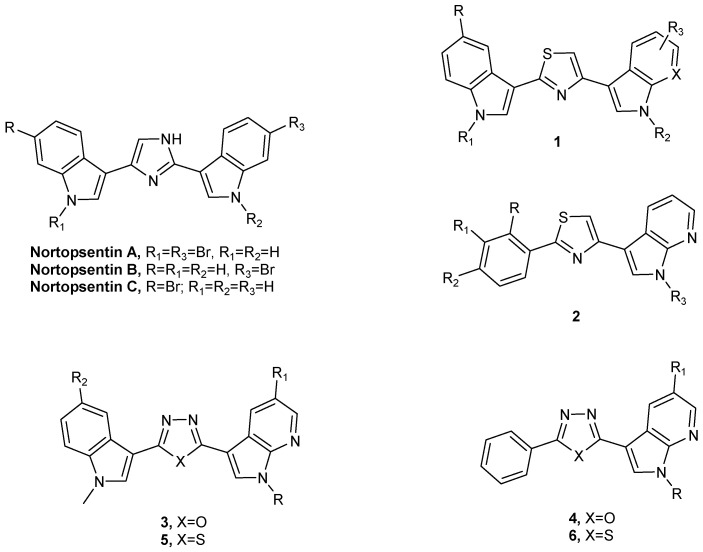
Structures of marine alkaloids Nortopsentins, their derivatives **1**,**2** and novel 1,3,4-oxadiazoles **3**,**5** and 1,3,4-thiadiazoles **4**,**6**.

Given the remarkable success of marine-derived molecules, our research team undertook the synthesis of a diverse compound library by modifying the structure of nortopsentins A–C (Figure 1), natural bis-indolyl alkaloids isolated from deep-sea sponge *Spongsorites ruetzleri*, having an imidazole as a spacer between the two indole units, with interesting antiproliferative activity against the murine leukemia cell line P388 (IC_50_ 4.5–20.7 μM). Analogs in which the imidazole ring of the alkaloid was replaced by other five- or six-membered heterocycles were assessed in the following studies [3,4,5,6,7,8,9,10,11,12]. Most of them showed antiproliferative activity against a wide range of human tumor cell lines, with the GI_50_ in the micromolar to the sub-micromolar range. The literature also reports numerous analogs where, in addition to the imidazole core, either one or both indole units were substituted with phenyl or azaindole. In particular, indolyl-thiazolyl-azaindoles **1** and **2** (Figure 1) demonstrated activity against a wide range of human tumor cell lines at micromolar and submicromolar concentrations, including STO and Meso II primary cell cultures derived from human diffuse malignant peritoneal mesothelioma (DMPM). In these cells, our compounds exhibited CDK1 inhibition with IC_50_ values ranging from 0.64 to 0.89 μM, comparable to the well-known CDK1 inhibitors roscovitine and purvalanol A. The activity of these compounds was further validated in an in vivo mouse model. The intraperitoneal administration of selected derivatives belonging to type **1** resulted in a substantial inhibition of tumor volume in DMPM xenografts (ranging from 58% to 75%) at doses that were well tolerated. Notably, two complete responses were observed in each treatment group [13].

Moreover, the treatment of colorectal cancer stem cells (CR-CSCs) with indolyl- 7-azaindolyl thiazoles **1** induced a reduction in CSC viability, making them sensitive to conventional chemotherapy drugs, such as oxaliplatin and 5- fluorouracil (5FU). In addition, the combination therapy of these derivatives with the CHK1 inhibitor rabusertinib showed a synergistic effect, abrogating CR-CSC’s proliferative and clonogenic potential [14].

Continuing our studies on nitrogen heterocyclic systems endowed with antitumor activity, herein, we report a new series of oxadiazole **3**,**5** and thiadiazole **4**,**6** nortopsentin derivatives (Figure 1), in which one indole portion was replaced with a 7-azaindole moiety or phenyl ring. These nitrogen heterocycles constitute the pharmacophore moieties of several molecules with different biological activities, including antitumor activity [9].

Hence, we comprehensively assessed the novel derivatives’ antitumor efficacy against various clinically relevant pancreatic ductal adenocarcinoma (PDAC) models. PDAC is an exceptionally aggressive malignancy projected to become the second-leading cause of cancer-related fatalities worldwide by 2040 [15]. The current treatment approach for most PDAC patients relies on cytotoxic chemotherapy, utilizing the combination of gemcitabine and nab-paclitaxel or the FOLFIRINOX regimen, consisting of 5-FU, leucovorin, irinotecan, and oxaliplatin, for unresectable cases. However, PDAC exhibits high resistance to chemotherapy, resulting in a dismal 5-year survival rate below 11%, underscoring the critical need for novel and more efficacious therapeutic options [16].

## 2. Results

### 2.1. Chemistry 

**Scheme 1:**
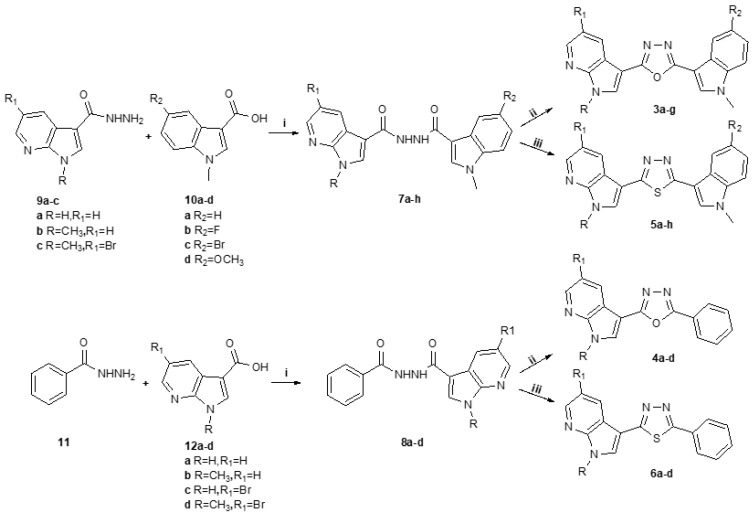
Synthesis of 1,3,4-oxadiazoles **3**,**4** and 1,3,4-thiadiazoles **5**,**6**. Reagents and conditions: (i) EDCI, HOBt, DMF, r.t 1–4 h, then r.t. 24 h or 60 °C 2–24 h or reflux 24 h; (ii) POCl_3_, reflux 3–6 h or 45° 24–48 h; (iii) Lewesson’s reagent, pyridine, reflux, 4–48 h.

The 1,3,4-oxadiazole compounds **3**,**4** and the 1,3,4-thiadiazoles **5**,**6** were synthesized from the key intermediates **7** or **8**, as shown in Scheme 1. The key intermediates **7** were prepared in very good yields (50–88%) through the reaction of hydrazines of type **9** and acids of type **10**, both commercially available, in the presence of 1-ethyl-3(3-dimethylaminopropyl) carbodiimide (EDCI), hydroxybenzotriazole (HOBt), and diisopropylethylamine (DIPEA) in dimethylformamide (DMF). The unavailable hydrazine **9c** was in turn synthesized from the corresponding carboxylic acid **10c** treated with hydrazine, according to the procedure already reported in the literature [17,18].

Compounds **7** were then cyclized using phosphoryl chloride or Lawesson’s reagent in pyridine under reflux, to obtain compounds **3** and **5**, respectively, in yields from good to excellent (Table 1). Analogously, compounds **4** and **6** were synthesized from commercially available benzoylhydrazine **11** and carboxylic acids **12**, reacted using the same conditions used for the synthesis of the key intermediates **7** already presented, to obtain the desired key intermediates **8** in excellent yields (91–95%). The latter were then cyclized in phosphoryl chloride or using Lawesson’s reagent in pyridine to afford compounds **4** and **6**, respectively, in good yields (Table 1).

### 2.2. Biology 

#### 2.2.1. Antiproliferative Activity 

**Figure 2:**
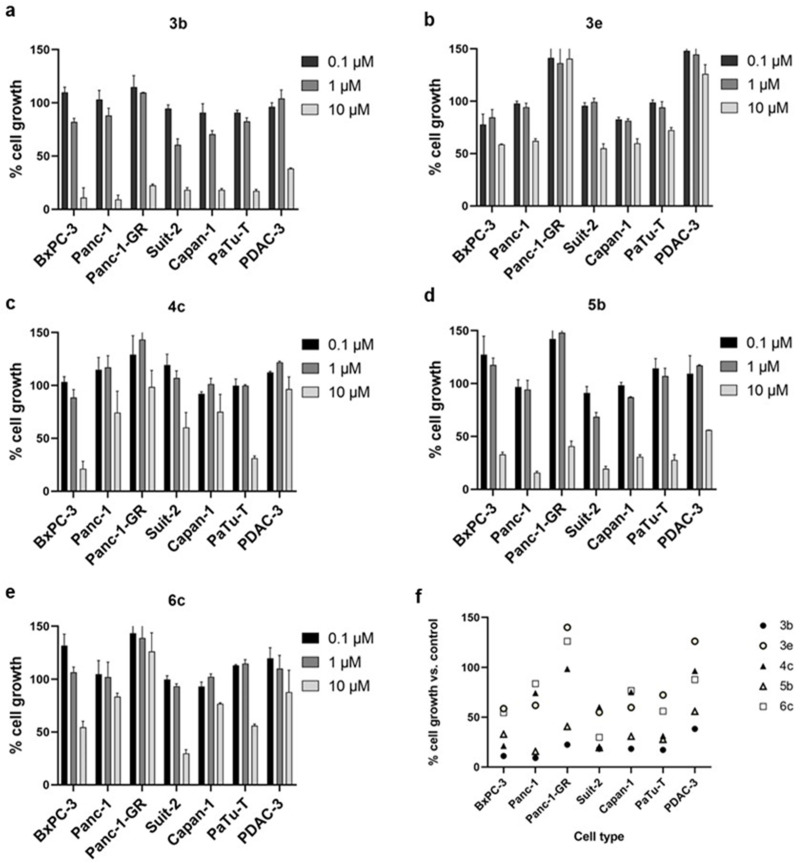
Cell growth after 72 h of treatment with each of the most active compounds **3b**, **3e**, **4c**, **5b**, **6c** (**a**–**e**) against each pancreatic cancer cell line exposed to three concentrations (0.1, 1 and 10 μM). (**f**) Cell growth data summary after 72 h of treatment with each of the most active compounds **3b**, **3e**, **4c**, **5b**, **6c** at a concentration of 10 μM. All the compounds showed significant antiproliferative activity against BxPC-3, Panc-1, Suit-2, Capan-1, and PaTu-T cells at the concentration of 10 μM. On the other hand, compounds **3b** and **5b** were the only active ones against PDAC-3 and Panc-1-GR The results are expressed as a percentage of cell growth compared to untreated control cells. Columns, mean values obtained from three independent experiments; bars, SEM.

The antiproliferative activity of compounds **3–6** was evaluated on a panel of immortalized cells of PDAC, a devastating type of cancer with poor survival rates due to very limited therapeutic options. In particular, the synthesized compounds were tested against BxPC-3, Panc-1, Suit-2, Capan-1, and PaTu-T cells, primary cell culture PDAC-3 and the gemcitabine-resistant cells Panc-1-GR, by Sulforhodamine-B (SRB) assay. In Figure 2, the results of the most active compounds **3b**, **3e**, **4c**, **5b**, **6c** are reported, showing the cell growth percentage after 72 h of exposure to three concentrations (0.1, 1, and 10 μM). All compounds showed significant antiproliferative activity against BxPC-3, Panc-1, Suit-2, Capan-1, and PaTu-T cells at the concentration of 10 μM. As expected, Panc-1-GR cells were less sensitive to all compounds compared to Panc-1 cells, at the same concentration (Table 2). However, compounds **3b** and **5b** were also active against PDAC-3 and Panc-1-GR cells at the concentration of 10 μM, with mean growth percentage values of 38.35% and 55.90% against PDAC-3, and 22.47% and 40.65% against Panc-1-GR cells, respectively. Conversely, compounds **3e** and **6c** did not determine growth inhibition against Panc-1-GR cells, and compound **3e** did not cause the growth inhibition of PDAC-3 cells (Table 2). These results may be related to an alteration of various cell signaling pathways, transcriptional factors and enzymes, due to the previous treatment with gemcitabine, which causes resistance in this cell type. Panc-1-GR cells have been shown to upregulate stem cell genes [19], and exhibit the overexpression of proteins, including Microtubule-associated protein 2 (MAP2), associated with poorer overall survival in patients treated with gemcitabine [20]. Despite these findings, the precise mechanisms responsible for the resistance to gemcitabine are still under investigation, and other gemcitabine-resistant variants showed high ITGA2 expression, highlighting the relevance of mechanical properties as potential therapeutic targets [21].

In order to more accurately determine the concentration at which cell growth was inhibited by 50% (IC_50_), PDAC cells were exposed to increasing concentrations of each compound for 72 h, ranging from 0.312 μM to 40 μM. These studies revealed that Suit-2 cells were the most sensitive cells to treatment with compounds **3b**, **3e**, and **5b** (with IC_50_ values of 1.4, 9.4, and 1.9 μM, respectively); meanwhile, BxPC-3 were sensitive to treatment with compound **4c** (IC_50_ value of 3.3 μM), and PaTu-T cells were sensitive to treatment with compound **6c** (IC_50_ value of 3.8 μM), respectively. In contrast, most compounds exhibited only weak antiproliferative activity against PDAC-3 and Panc-1-GR cells, for which the only active compounds were **3b** and **5b**, with IC_50_ values of 8.1 and 10.6 μM against PDAC-3 cells, and 8.0 and 9.8 μM against Panc-1-GR cells, respectively. Among all the compounds tested, the most active ones from each series were 1,3,4-oxadiazole **3b** (with IC_50_ values ranging from 1.4 to 8.1 μM) and 1,3,4-thiadiazole **5b** (with IC_50_ values ranging from 1.9 to 10.6 μM), as shown in Table 3.

**Figure 3:**
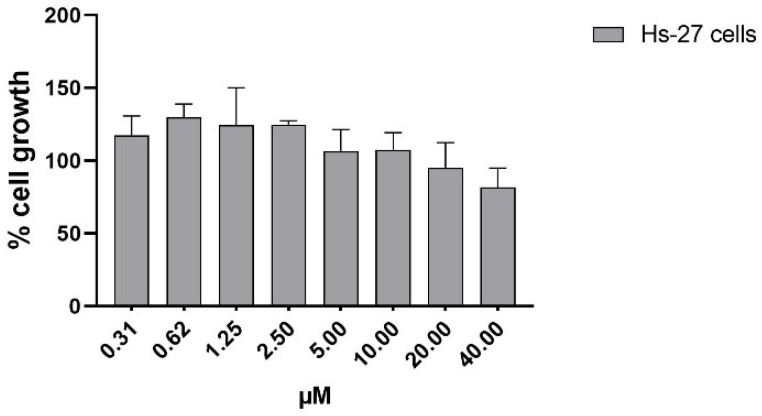
Evaluation of the inhibition of cell growth/cytotoxicity of compound **3b** at different concentrations (from 0.31 μM to 40 μM) against normal human skin fibroblast Hs27 cells. As shown, the derivative was relatively non-toxic to normal fibroblast cells. Columns, mean values obtained from three independent experiments; bars, SEM.

Additional experiments were conducted to evaluate the in vitro cytotoxicity of the most active compound, **3b**, against normal human skin fibroblasts Hs27. The compound was tested at eight different concentrations ranging from 0.31 μM to 40 μM using the SRB assay. The results demonstrated that this compound was relatively non-toxic towards normal fibroblast cells. At the highest concentration tested (40 μM), only 18% growth inhibition was observed after 72 h of exposure. Furthermore, concentrations below 10 μM showed cell growth rates around or slightly exceeding 100%, similar to untreated cells, as illustrated in Figure 3, supporting the absence of cytotoxicity in human normal cells. These findings provide evidence of the potential safety of compound **3b** for further development as a therapeutic agent.

#### 2.2.2. Effects on Cell Cycle

**Figure 4:**
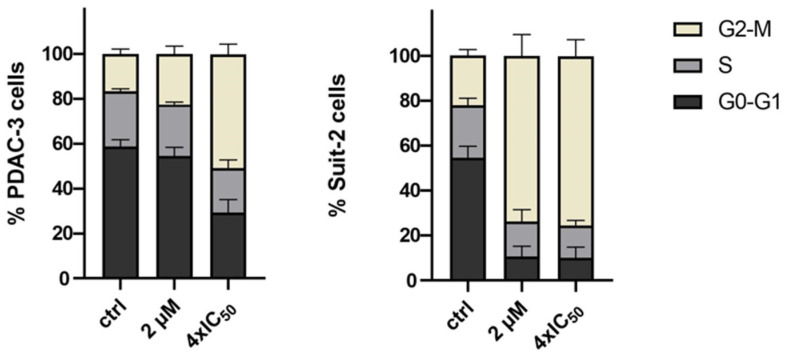
Effects of the oxadiazole compound **3b** on cell cycle modulation in PDAC-3 cells and in Suit-2 cells. Cells were exposed for 24 h to this compound. Columns show the mean percentages of cells at various stages of the cell cycle, G0-G1 (black), S (dark grey), and G2-M (light grey) phase, in the untreated control (ctrl) and after treatment with the compound at concentrations of 2 μM and 4-fold IC_50_. Cell-cycle analysis showed an increase in G2-M cells and a decrease in the G1 phase. Columns, mean values obtained from three independent measurements; bars, SEM.

The effects of the most active compound **3b** on cell cycle progression were investigated in primary cell culture PDAC-3, which was relatively more resistant, and in the Suit-2 cells, which exhibited the highest sensitivity to this compound. For cytofluorimetric analysis of cell cycle alterations, a propidium iodide (PI) staining solution was utilized. The results revealed that compound **3b** induced changes in the distribution of cells across the cell-cycle phases. PDAC-3 cells treated with 2 μM and 4-fold IC_50_ concentrations of **3b** exhibited a decrease in the percentage of cells in the G0-G1 phase from 59% to 54% and 29%, respectively. Similarly, the percentage of cells in the S phase decreased from 25% to 23% and 19%. In contrast, there was an increase in the percentage of cells in the G2-M phase from 16% to 23% and 52%. Suit-2 cells demonstrated a more pronounced cell cycle arrest in the G2-M phase. Treatment with 2 μM and 4-fold IC_50_ concentrations of compound **3b** resulted in a decrease in the percentage of cells in the G0-G1 phase from 54% to 10% and 10%, respectively. Furthermore, the percentage of cells in the S phase decreased from 23% to 15% and 14%. Conversely, there was a significant increase in the percentage of cells in the G2-M phase, from 23% to 75% and 76%. These findings, as shown in Figure 4, suggest that compound **3b** may exert its effect by inhibiting protein kinases involved in the cell cycle process.

#### 2.2.3. Anti-Migratory Activity

**Figure 5:**
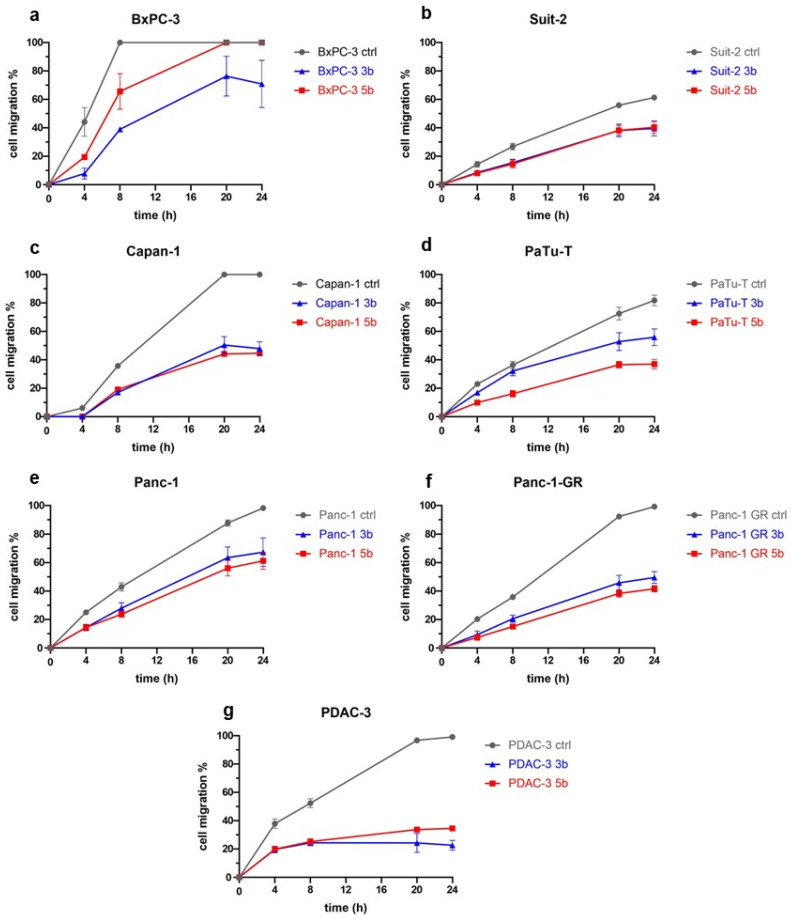
Cell migration percentage monitored over time (0, 4, 8, 20 and 24 h) of BxPC-3 (**a**), Suit-2 (**b**), Capan-1 (**c**), PaTu-T (**d**), Panc-1 (**e**), Panc-1-GR (**f**), and PDAC-3 (**g**) cells treated with compounds **3b** and **5b** at a concentration of 4 × IC_50_. As shown, compounds **3b** and **5b** showed marked selective antimigratory activity against PDAC-3, Panc-1-GR and Capan-1 cells, compared to the untreated controls (set at 100%). Points, mean values obtained from the means of at least three different scratch areas.

The aggressiveness of PDAC is closely associated with cell migration, which plays a crucial role in metastasis [22]. Therefore, there is a pressing need for novel compounds capable of inhibiting cell migration and preventing metastatic spread in PDAC. To investigate the effect of the most active compounds **3b** and **5b** on cell migration in vitro, we performed the scratch wound healing assay on seven PDAC cell models (BxPC-3, Suit-2, Capan-1, PaTu-T, Panc-1, Panc-1-GR, and PDAC-3) using a 96-pin floating array device. Our results (Figure 5) showed that both compounds inhibited cell migration compared to untreated cells as early as 4 h and continued up to 24 h in most PDAC cell lines tested, except for BxPC-3 and Capan-1 cells. However, BxPC-3 cells showed a reduction in cell migration after 8 h of treatment with compounds **3b** and **5b**, with only 39% and 66% of cells migrating, respectively, compared to untreated cells that achieved 100% wound closure. On the other hand, Capan-1 cells showed an anti-migratory effect from 8 h up to 24 h, with no effect observed before 4 h.

Moreover, compounds **3b** and **5b** displayed notable anti-migratory activity against PDAC-3, Panc-1-GR, and Capan-1 cell lines compared to the (untreated) controls, with migration percentages of cells treated with the compounds ranging from 23% to 48% after 24 h. In other cell lines, we observed an inhibitory effect on cell migration at 24 h, with the percentage of migrated cells treated with each compound ranging from 45% to 100% compared to the control. Overall, these data suggest that compounds **3b** and **5b** have the potential to significantly reduce the rate of cell migration in various preclinical models of PDAC, with compound **3b** demonstrating the highest effectiveness.

#### 2.2.4. Modulation of CDK1 Expression by ELISA

**Figure 6:**
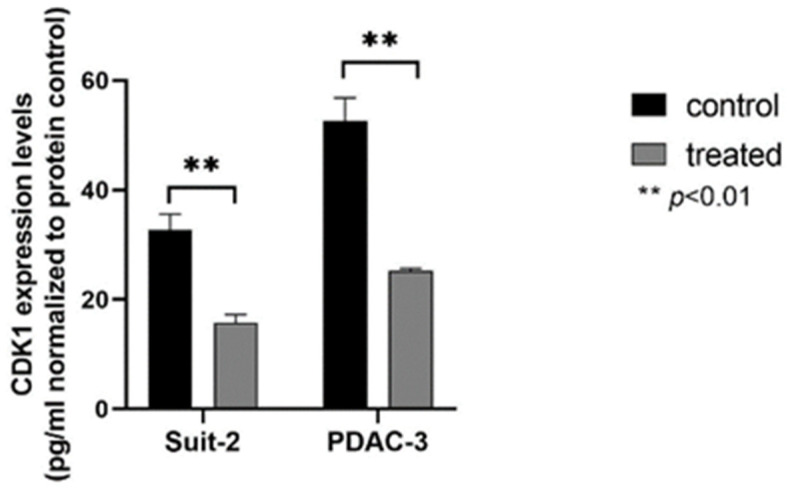
ELISA-based quantitation of CDK1 expression in PDAC cells following a 24 h exposure to DMSO (control) or to 10 μM of compound **3b.** CDK1 expression was quantified using a human cyclin-dependent kinase 1 (CDK1) ELISA Kit according to the manufacturer’s instructions. As shown, a reduction in CDK1 expression in both cellular models compared to control cells was observed. Columns: mean; bars: SEM (*n* = 3). **: *p* < 0.01.

**Figure 7:**
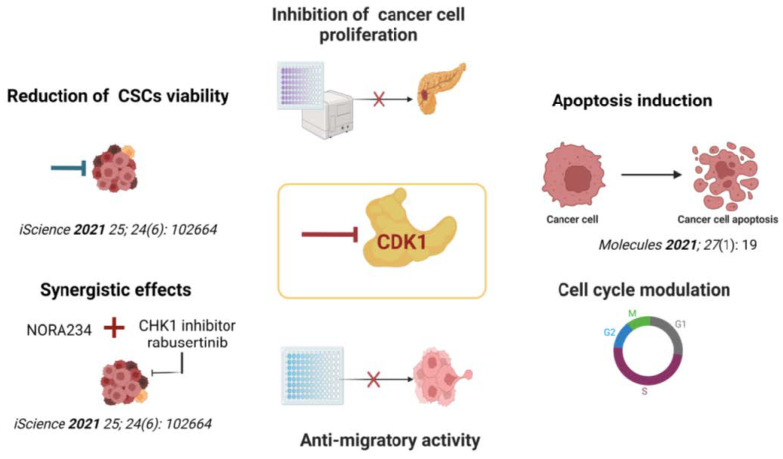
Schematic illustration of the potential regulatory pathway of compound **3b** against PDAC cells, based on these results or previous studies [10,14].

The findings from our previous studies suggested that compound **3b** may exhibit its anti-cancer effects by targeting CDK1 expression [23]. To investigate this further, we conducted a specific ELISA assay to assess CDK1 expression in two PDAC preclinical models (Suit-2 and PDAC-3) treated with 10 µM of compound **3b**. The results showed a reduction in CDK1 expression in both models compared to control cells, indicating that compound **3b** may target CDK1 to exert its anti-cancer effects (Figure 6). This observation suggests that compound **3b** may specifically interact with CDK1, potentially serving as the mechanism behind its anti-cancer effects. These results, as depicted in Figure 7, provide valuable insights into the potential mode of action of compound **3b** and strengthen its prospects as a therapeutic agent for PDAC treatment.

## 3. Materials and Methods

### 3.1. Chemistry

Analytical thin-layer chromatography (TLC) was performed on silica gel 60 F254 plates (0.25 mm thickness) and the developed plates were examined under ultraviolet (UV) light. All melting points were taken on a Buchi-Tottoly capillary apparatus and were uncorrected. IR spectra were determined in bromoform with a Shimadzu FT/IR 8400S spectrophotometer and peaks were reported in wavenumber (cm^−1^). ^1^H and ^13^C NMR spectra were measured at 200 and 50 MHz, respectively, on DMSO-d_6_ solution, using a Bruker Avance II series 200 MHz spectrometer. The chromatography column was performed with MERK silica gel 230–400 mesh ASTM or FLASH40i Biotage chromatography or with a Buchi Sepacore chromatography module (prepacked cartridge reference). Elementary analyses (C, H, N) were within ± 0.4% of the theoretical values and were performed with a VARIO EL III elemental analyzer (Elementar, Langenselbold, Germany). Compounds **3–6**,**7c**,**8b** were characterized only by ^1^H NMR spectra, for their poor solubility.

#### 3.1.1. General Procedure for the Synthesis of 5-Bromo-1-methyl-7-azaindole-3-carbohydrazide (**9c**)

Appropriate carboxylic acid **10** (3.1 mmol) was stirred at room temperature with thionyl chloride (68 mmol), then the reaction mixture was heated under reflux for three hours. The reaction mixture was cooled and the excess of thionyl chloride (SOCl_2_) was distilled off. The resulting solid was recrystallized from aqueous ethanol, dissolved in absolute alcohol and heated under reflux with hydrazine hydrate to give 7-azaindole-3-carbohydrazide. The product was then recrystallized from aqueous ethanol.

Yield: 70%; white solid; mp: 130 °C; IR (cm^−1^): 3310 (NH_2_), 3148 (NH), 1684 (CO); ^1^H NMR (200 MHz, DMSO-*d_6_*) δ: 9.40 (bs, 1H, NH), 8.58 (d, *J* = 2.3 Hz, 1H), 8.42 (d, *J* = 2.2 Hz, 1H), 8.17 (s, 1H), 4.40 (s, 2H, NH_2_), 3.84 (s, 3H, CH_3_); ^13^C NMR (50 MHz, DMSO-*d_6_*) δ: 164.7 (s), 148.5 (s), 144.6 (d), 134.3 (d), 130.1 (d), 120.0 (s), 111.3 (s), 109.4 (s), 31.6 (q); Anal. Calculated for C_9_H_9_BrN_4_O (MW: 269,10): C, 40.17; H, 3.37; N, 20.82%. Found: C, 40.17; H, 3.37; N, 20.82%.

#### 3.1.2. General Procedure for the Synthesis of N’-(1-Methyl-1H-indole-3-carbonyl)-7-azaindole-3-carbohydrazides (**7a–h**)

To a solution of the proper acid **10a–d** (2 mmol) in dimethylformamide (10 mL), 1-ethyl-3(3-dimethylaminopropyl)carbodiimide (2 mmol), hydroxylbenzotriazole (2 mmol) and diisopropylethylamine (4 mmol) were added. The reaction was stirred for 1–4 h at room temperature. Then, the proper hydrazine **9a–c** (4 mmol) was added, and the reaction mixture was heated at 60° for 2–24 h or under reflux for 24 h. Once cooled, ice was added, and the formed precipitate was filtered off and recrystallized in diethyl ether to afford the pure compounds.

*N’-(1-methyl-1H-indole-3-carbonyl)-7-azaindole-3-carbohydrazide* (**7a**). Conditions: r.t. for 1 h, then reflux for 24 h. Yield: 50%; brown solid; mp: 182 °C; IR (cm^−1^): 3447 (NH), 3366 (NH), 3229 (NH), 1558 (CO), 1541 (CO); ^1^H NMR (200 MHz, DMSO-*d_6_*) δ: 12.21 (bs, 1H, NH), 9.94 (bs, 1H, NH), 9.83 (bs, 1H, NH), 8.44 (d, *J* = 6.7 Hz, 1H), 8.32–8.25 (m, 2H), 8.15 (d, *J* = 7.9 Hz, 1H), 8.11 (s, 1H), 7.53 (d, *J* = 8.2 Hz, 1H), 7.28–7.22 (m, 1H), 7.22– 7.19 (m, 1H), 7.18 (dd, *J* = 10.0, 5.2 Hz, 1H), 3.87 (s, 3H, CH_3_); ^13^C NMR (101 MHz, DMSO-*d_6_*) δ: 148.8 (s), 144.2 (d), 137.1 (s), 132.6 (d), 129.7 (d), 129.0 (d), 127.1 (s), 126.2 (s), 122.6 (d), 121.5 (d), 121.3 (d), 119.8 (s), 119.1 (s), 117.6 (d), 110.8 (d), 107.9 (s), 107.2 (s), 33.6 (q); *Anal.* Calculated for C_18_H_15_N_5_O_2_ (MW: 333,35): C, 64.86; H, 4.54; N, 21.01%. Found C, 64.71; H, 4.50; N, 21.22%.

*1-methyl-N’-(1-methyl-1H-indole-3-carbonyl)-7-azaindole-3-carbohydrazide* (**7b**). Conditions: r.t. for 4 h, then 60 °C.for 2 h. Yield: 80%; brown solid; mp: 159 °C; IR (cm^−1^): 3228 (2 NH), 1653 (CO), 1636 (CO); ^1^H NMR (200 MHz, DMSO-*d_6_*) δ: 9.98 (bs, 1H, NH), 9.87 (bs, 1H, NH), 8.49–8.45 (dd, *J* = 1.4, 7.9 Hz, 1H), 8.39–8.36 (dd, *J* = 1.4, 4.6 Hz, 1H), 8.31 (s, 1H), 8.18–8.15 (d, *J* = 7.3 Hz, 1H), 8.13 (s, 1H), 7.56–7.52 (d, *J* = 8 Hz, 1H), 7.29–7.15 (m, 3H), 3.91 (s, 3H, CH_3_), 3.88 (s, 3H, CH_3_); ^13^C NMR (50 MHz, DMSO-*d_6_*) δ: 163.6 (s), 147.3 (s), 143.5 (d), 136.6 (s), 132.1 (d), 129.4 (d), 126.6 (s), 122.1 (d), 121.0 (d), 120.9 (dx2), 118.8 (s), 117.2 (d), 110.3 (d), 107.3 (sx2), 106.2 (s), 33.1 (q), 31.4 (q); *Anal.* Calculated for C_19_H_17_N_5_O_2_ (MW: 347,38): C, 65.69; H, 4.93; N, 20.16%. Found C, 65.57; H, 4.81; N, 20.10%.

*N’-(5-fluoro-1-methyl-1H-indole-3-carbonyl)-1-methyl-7-azaindole-3-carbohydrazide* (**7c**). Conditions: r.t. for 2 h, then 60 °C for 24 h. Yield: 85%; brown solid; mp: 172 °C; IR (cm^−1^): 3184 (NH), 3119 (NH), 1576 (CO), 1526 (CO); ^1^H NMR (200 MHz, DMSO-*d_6_*) δ: 9.99 (bs, 1H, NH), 9.93 (bs, 1H, NH), 8.48–8.44 (d, *J* = 7.5 Hz, 1H), 8.38–8.36 (d, *J* = 4.6 Hz, 1H), 8.31 (s, 1H), 8.19 (s, 1H), 7.86–7.80 (dd, *J* = 2.3, 10 Hz, 1H), 7.61–7.54 (dd, *J* = 4.3, 8.9 Hz, 1H), 7.29–7.22 (dd, *J* = 4.7, 7.9 Hz, 1H), 7.17–7.07 (td, *J* = 2.4, 9.2 Hz, 1H), 3.91 (s, 3H, CH_3_), 3.89 (s, 3H, CH_3_); *Anal.* Calculated for C_19_H_16_FN_5_O_2_ (MW: 365,37): C, 62.46; H, 4.41; N, 19.17%. Found: C, 62.40; H, 4.37; N, 19.22%.

*N’-(5-bromo-1-methyl-1H-indole-3-carbonyl)-1-methyl-7-azaindole-3-carbohydrazide* (**7d**). Conditions: r.t. for 1 h, then 60 °C for 24 h. Yield: 88%; white solid; mp: 169 °C; IR (cm^−1^): 3184–3199 (2 NH), 1647 (CO), 1578 (CO); ^1^H NMR (200 MHz, DMSO-*d_6_*) δ: 9.99 (bs, 1H, NH), 9.96 (bs, 1H, NH), 8.48–8.44 (d, *J* = 7.8 Hz, 1H), 8.39–8.36 (d, *J* = 4.6 Hz, 1H), 8.31 (s, 2H), 8.17 (s, 1H), 7.57–7.52 (d, *J* = 8.7 Hz, 1H), 7.41–7.36 (dd, *J* = 1.7, 8.8 Hz, 1H), 7.29–7.22 (dd, *J* = 4.7, 7.8 Hz, 1H), 3.91 (s, 3H, CH_3_), 3.88 (s, 3H, CH_3_); ^13^C NMR (50 MHz, DMSO-*d_6_*) δ: 163.7 (s), 163.5 (s), 147.3 (s), 143.5 (d), 135.4 (s), 133.3 (d), 132.2 (d), 129.4 (d), 128.3 (s), 124.7 (d), 123.1 (d), 118.8 (s), 117.3 (d), 113.8 (s), 112.6 (d), 106.9 (s), 106.1 (s), 33.3 (q), 31.3 (q); *Anal.* Calculated for C_19_H_16_BrN_5_O_2_ (MW: 426,27): C, 53.54; H, 3.78; N, 16.43%. Found: C, 53.50; H, 3.74; N, 16.33%.

*5-bromo-1-methyl-N’-(1-methyl-1H-indole-3-carbonyl)-7-azaindole-3-carbohydrazide* (**7e**). Conditions: r.t. for 4 h, then 60 °C.for 2 h. Yield: 80%; white solid; mp: 175 °C; IR (cm^−1^): 3354 (NH), 3254 (NH), 1558 (CO), 1541 (CO); 1H NMR (200 MHz, DMSO-*d_6_*) δ: 9.91–9.96 (bs, 2H, 2NH), 8.61–8.60 (d, *J* = 2.2 Hz, 1H), 8.46–8.45 (d, *J* = 2.2 Hz, 1H), 8.37 (s, 1H), 8.18–8.13 (d, *J* = 9.0 Hz, 1H), 8.13 (s, 1H), 7.56–7.52 (d, *J* = 7.6 Hz, 1H), 7.30–7.15 (m, 2H), 3.91 (s, 3H, CH_3_), 3.88 (s, 3H, CH_3_); ^13^C NMR (50 MHz, DMSO-*d_6_*) δ: 163.1 (s), 162.3 (s), 145.8 (s), 143.6 (d), 136.6 (s), 133.8 (d), 132.1 (d), 131.1 (d), 126.6 (s), 122.1 (d), 121.0 (d), 120.9 (d), 120.3 (s), 112.7 (s), 110.3 (d), 107.2 (s), 105.8 (s), 33.1 (q), 31.6 (q); *Anal.* Calculated for C_19_H_16_BrN_5_O_2_ (MW: 426,27): C, 53.54; H, 3.78; N, 16.43%. Found: C, 53.50; H, 3.81; N, 16.39%.

*5-bromo-N’-(5-bromo-1-methyl-1H-indole-3-carbonyl)-1-methyl-7-azaindole-3-carbohydrazide* (**7f**). Conditions: r.t. for 1 h, then 60 °C for 6 h; Yield: 82%; white solid; mp: 168 °C; IR (cm^−1^): 3244 (2 NH), 1730 (2 CO); ^1^H NMR (200 MHz, DMSO-*d_6_*) δ: 10.0 (bs, 1H, NH), 9.93 (bs, 1H, NH), 8.49–8.44 (dd, *J* = 1.5, 7.9 Hz, 1H), 8.38–8.35 (dd, *J* = 1.5, 4.7 Hz, 1H), 8.20 (s, 1H), 7.87–7.81 (dd, *J* = 2.5, 10 Hz, 1H), 7.61–7.54 (dd, *J* = 4.5, 8.9 Hz, 1H), 7.29–7.23 (dd, *J* = 4.7, 7.9 Hz, 1H), 7.17–7.07 (td, *J* = 2.6, 9.2 Hz, 1H), 3.91 (s, 3H, CH_3_), 3.89 (s, 3H, CH_3_); ^13^C NMR (50 MHz, DMSO-*d_6_*) δ: 163.3 (s), 162.4 (s), 145.7 (s), 143.5 (d), 133.61 (d), 132.2 (d), 130.7 (s), 129.4 (d), 127.3 (s), 120.1 (s), 117.3 (d), 112.4 (s), 110.1 (s), 108.9 (s), 105.9 (d), 105.4 (d), 104.8 (s), 33.4 (q), 31.4 (q); *Anal.* Calculated for C_19_H_15_Br_2_N_5_O_2_ (MW: 505,17): C, 45.17; H, 2.99; N, 13.86%. Found C, 45.12; H, 2.88; N, 13.80%.

*5-bromo-N’-(5-fluoro-1-methyl-1H-indole-3-carbonyl)-1-methyl-7-azaindole-3-carbohydrazide* (**7g**). Conditions: r.t. for 3 h, then 60 °C for 24 h. Yield: 85%; brown solid; mp: 189 °C; IR (cm^−1^): 3420 (NH), 3312 (NH), 1653 (CO), 1616 (CO); ^1^H NMR (200 MHz, DMSO-*d_6_*) δ: 10.08 (bs, H, NH), 9.96 (bs, H, NH), 8.60–8.59 (d, *J* = 2.1 Hz, 1H), 8.46–8.45 (d, *J* = 2.2 Hz, 1H), 8.36 (s, 1H), 8.19 (s, 1H), 7.86–7.79 (dd, *J* = 2.4, 10.0 Hz, 1H), 7.61–7.55 (dd, *J* = 4.5, 8.9 Hz, 1H), 7.17–7.07 (td, *J* = 2.5, 9.2 Hz, 1H), 3.90 (s, 3H, CH_3_), 3.89 (s, 3H, CH_3_); ^13^C NMR (50 MHz, DMSO-*d_6_*) δ: 164.3 (s), 163.6 (s), 159.8 (d, *J_C-F_* =114.2 Hz), 146.3 (s), 144.1 (d), 134.4 (s), 134.3 (d, *J_C-F_* =20.7 Hz), 133.9 (s), 131.6 (s), 120.3 (s), 120.8 (s), 113.2 (s), 112.4 (d), 112.3 (d), 110.9 (d, *J_C-F_* =26.4 Hz), 106.2 (d, *J_C-F_* =24.5 Hz), 106.1 (s), 33.9 (q), 32.1 (q); Anal. Calculated for C_19_H_15_BrFN_5_O_2_ (MW: 444,26): C, 51.37; H, 3.40; N, 15.76%. Found: C, 51.30; H, 3.47; N, 15.79%.

*N’-(5-methoxy-1-methyl-1H-indole-3-carbonyl)-1-methyl-7-azaindole-3-carbohydrazide* (**7h**). Conditions: r.t. for 4 h, then 60 °C for 24 h. Yield: 88%; white solid; mp: 198 °C; IR (cm^−1^): 3231–3101 (2 NH), 1624 (CO), 1541 (CO); ^1^H NMR (200 MHz, DMSO-*d_6_*) δ: 9.94 (bs, 1H, NH), 9.82 (bs, 1H, NH), 8.48–8.45 (d, *J* = 6.9 Hz, 1H), 8.38–8.36 (d, *J* = 4.5 Hz, 1H), 8.30 (s, 1H), 8.06 (s, 1H), 7.67–7.66 (d, *J* = 2.3 Hz, 1H), 7.46–7.42 (d, *J* = 8.9 Hz, 1H), 7.29–7.23 (dd, *J* = 4.7, 7.8 Hz, 1H), 6.91–6.85 (dd, *J* = 2.4, 8.9 Hz, 1H), 3.91 (s, 3H, OCH_3_), 3.84 (s, 3H, CH_3_), 3.77 (s, 3H, CH_3_); ^13^C NMR (50 MHz, DMSO-*d_6_*) δ: 162.2 (s), 150.1 (s), 143.5 (d), 132.2 (d), 132.1 (d), 130.2 (s), 129.4 (d), 124.4 (s), 120.1 (s), 117.3 (d), 115.4 (s), 112.2 (d), 111.2 (d), 110.2 (s), 105.6 (s), 102.5 (d), 101.9 (s), 55.2 (q), 32.2 (q), 31.4 (q); *Anal.* Calculated for C_20_H_19_N_5_O_3_ (MW: 377,40): C, 63.65; H, 5.07; N, 18.56%. Found: C, 63.55; H, 5.21; N, 18.60%.

#### 3.1.3. General Procedure for the Synthesis of N’-Benzoyl-7-azaindole-3-carbohydrazides (**8a–d**)

To a solution of the proper acid **12** (2 mmol) in dimethylformamide (10 mL), 1-ethyl-3(3-dimethylaminopropil)carbodiimide (2 mmol), hydroxylbenzotriazole (2 mmol) and diisopropylethylamine (4 mmol) were added. The reaction was stirred for 1 h at room temperature. Then, the proper hydrazine **11** (4 mmol) was added and the reaction mixture was heated at room temperature for 24 h. Once cooled, ice was added and the formed precipitate was filtered off and recrystallized in diethyl ether to afford the pure compounds.

*N’-benzoyl-7-azaindole-3-carbohydrazide* (**8a**). Yield: 92%; white solid; mp: 170 °C; IR (cm^−1^): 3454 (NH), 3377 (NH), 3221 (NH), 1636 (CO), 1558 (CO); ^1^H NMR (200 MHz, DMSO-*d_6_*) δ: 12.27 (bs, 1H, NH), 10.44 (bs, 1H, NH), 10.11 (bs, 1H, NH), 8.45 (d, *J* = 7.6 Hz, 1H), 8.30 (s, 2H), 7.95 (d, *J* = 6.8 Hz, 2H), 7.57 (t, *J* = 8.0 Hz, 3H), 7.22 (dd, *J* = 7.5, 4.8 Hz, 1H); ^13^C NMR (50 MHz, DMSO-*d_6_*) δ: 166.1 (s), 163.5 (s), 148.3 (s), 143.7 (d), 132.7 (s), 131.8 (d), 129.1 (d), 128.6 (d), 128.5 (dx2), 127.4 (dx2), 118.5 (s), 117.2 (d), 107.1 (s); *Anal.* Calculated for C_15_H_12_N_4_O_2_ (MW: 280,29): C, 64.28; H, 4.32; N, 19.99%. Found C, 64.32; H, 4.45; N, 19.80%.

*N’-benzoyl-1-methyl-7-azaindole-3-carbohydrazide* (**8b**). Yield: 94%; white solid; mp: 174 °C; IR (cm^−1^): 3219–3125 (2 NH), 1663 (CO), 1636 (CO); ^1^H NMR (200 MHz, DMSO-*d_6_*) δ: 10.44 (bs, 1H, NH), 10.16 (bs, 1H, NH), 8.58 (d, *J* = 1.5 Hz, 1H), 8.34 (s, 1H), 7.94 (s, 1H), 7.91 (d, *J* = 1.5 Hz, 2H), 7.60–7.58 (m, 1H), 7.54–7.51 (m, 3H), 3.89 (s, 3H, CH_3_); *Anal.* Calculated for C_16_H_14_N_4_O_2_ (MW: 294,31): C, 65.30; H, 4.79; N, 19.04%. Found C, 65.41; H, 4.82; N, 19.00%.

*N’-benzoyl-5-bromo-7-azaindole-3-carbohydrazide* (**8c**). Yield: 92%; white solid; mp: 205 °C; IR (cm^−1^): 3458 (NH), 3184 (NH), 3098 (NH), 1628 (CO), 1558 (CO); ^1^H NMR (200 MHz, DMSO-*d_6_*) δ: 12.52 (bs, 1H, NH), 10.43 (bs, 1H, NH), 10.16 (bs, 1H, NH), 8.57 (d, *J* = 2.0 Hz, 1H), 8.39 (d, *J* = 2.0 Hz, 1H), 8.34 (s, 1H), 7.93 (d, *J* = 7.4 Hz, 2H), 7.60–7.51 (m, 3H); ^13^C NMR (50 MHz, DMSO-*d_6_*) δ: 166.6 (s), 147.2 (s), 144.4 (s), 133.2 (s), 132.3 (d), 131.4 (d), 130.8 (d), 129.0 (dx2), 127.9 (dx2), 127.8 (d), 120.7 (s), 113.0 (s), 107.3 (s); *Anal.* Calculated for C_15_H_11_BrN_4_O_2_ (MW: 359,18): C, 50.16; H, 3.09; N, 15.60%. Found C, 50.26; H, 3.21; N, 15.64%.

*N’-benzoyl-5-bromo-1-methyl-7-azaindole-3-carbohydrazide* (**8d**). Yield: 95%; white solid; mp: 180 °C; IR (cm^−1^): 3221 (NH), 3098 (NH), 1707 (CO), 1629 (CO); ^1^H NMR (200 MHz, DMSO-*d_6_*) δ: ^1^H NMR (200 MHz, DMSO-*d_6_*) δ: 10.47 (bs, 1H, NH), 10.21 (bs, 1H, NH), 8.59 (s, 1H), 8.47 (s, 1H), 8.36 (s, 1H), 7.94 (d, *J* = 7.4 Hz, 3H), 7.56 (d, *J* = 7.5 Hz, 2H), 3.91 (s, 3H, CH_3_); ^13^C NMR (50 MHz, DMSO-*d_6_*) δ: 166.5 (s), 163.4 (s), 146.3 (s), 144.2 (d), 138.9 (s), 134.5 (d), 132.3 (d), 131.6 (d), 129.0 (dx2), 127.9 (dx2), 120.7 (s), 113.3 (s), 106.1 (s), 32.1 (q); *Anal.* Calculated for C_16_H_13_BrN_4_O_2_ (MW: 373,21): C, 51.49; H, 3.51; N, 15.01%. Found C, 51.55; H, 3.61; N, 15.23%.

#### 3.1.4. General Procedure for the Synthesis of 3-[5-(1-Methyl-1H-indol-3-yl)-1,3,4-oxadiazol-2-yl]-7-azaindoles (**3a–g**)

The solution of the proper intermediate **7** (0.81 mmol) in phosphoryl chloride (5.4 mL) was heated at 45 °C for 24 or 48 h or under reflux for 3–6 h. Once cooled, the reaction mixture was evaporated in vacuo and extracted with a saturated solution of sodium hydrogen carbonate (3 × 30 mL) and ethyl acetate (3 × 30 mL). The organic layers were evaporated in vacuo and dried with sodium sulphate. The resulting crude material was purified in a column using dichloromethane/methanol 99:1 as eluent.

*3-[5-(1-methyl-1H-indol-3-yl)-1,3,4-oxadiazol-2-yl]-7-azaindole* (**3a**). Conditions: reflux for 4 h. Yield 75%; white solid; mp: 302 °C; IR (cm^−1^): 3117 (NH); ^1^H NMR (200 MHz, DMSO-*d_6_*) δ: 12.57 (s, 1H, NH), 8.53 (dd, *J* = 7.9, 1.6 Hz, 1H, Ar-H), 8.41 (dd, *J* = 4.7, 1.6 Hz, 1H, Ar-H), 8.37 (d, *J* = 2.4 Hz, 1H, Ar-H), 8.29 (s, 1H, Ar-H), 8.23–8.20 (m, 1H, Ar-H), 7.37–7.32 (m, 2H, Ar-H), 7.32–7.28 (m, 2H, Ar-H), 3.93 (s, 3H, CH_3_); *Anal.* Calculated for C_18_H_13_N_5_O (MW: 315,34): C, 68.56; H, 4.16; N, 22.21%. Found: C, 68.59; H, 4.21; N, 22.15%.

*1-methyl-3-[5-(1-methyl-1H-indol-3-yl)-1,3,4-oxadiazol-2-yl]-7-azaindole* (**3b**). Conditions: reflux for 6 h. Yield 86%; white solid, mp: 239 °C; ^1^H NMR (200 MHz, DMSO-*d6*) δ: 8.55–8.45 (m, 3H, 3 Ar-H), 8.30 (s, 1H, Ar-H), 8.23–8.19 (d, *J* = 7.3 Hz, 1H, Ar-H), 7.65–7.61 (d, *J* = 8.1 Hz, 1H, Ar-H), 7.51–7.37 (m, 3H, 3 Ar-H), 3.96 (s, 3H, CH_3_), 3.94 (s, 3H, CH_3_); *Anal.* Calculated for C_19_H_15_N_5_O (MW: 329,36): C, 69.29; H, 4.59; N, 21.26%. Found: C, 71.29; H, 4.69; N, 21.20%.

*3-[5-(5-fluoro-1-methyl-1H-indol-3-yl)-1,3,4-oxadiazol-2-yl]-1-methyl-7-azaindole* (**3c**). Conditions: 45 °C for 48 h. Yield 85%; white solid; mp: 268 °C; ^1^H NMR (200 MHz, DMSO-*d_6_*) δ: 8.56–8.53 (d, *J* = 7.1 Hz, 2H, 2 Ar-H), 8.48–8.46 (d, *J* = 4.0 Hz, 1H, Ar-H), 8.39 (s, 1H, Ar-H), 7.90–7.85 (d, *J* = 9.4 Hz, 1H, Ar-H), 7.71–7.65 (dd, *J* = 4.4, 8.9 Hz, 1H, Ar-H), 7.42–7.35 (dd, *J* = 5.1, 7.6 Hz, 1H, Ar-H), 7.28–7.24 (d, *J* = 8.8 Hz, 1H, Ar-H), 3.97 (s, 6H, 2 CH_3_); Anal. Calculated for C_19_H_14_FN_5_O (MW: 347,35): C, 65.70; H, 4.06; N, 20.16%. Found: C, 65.75; H, 4.13; N, 20.26%.

*3-[5-(5-bromo-1-methyl-1H-indol-3-yl)-1,3,4-oxadiazol-2-yl]-1-methyl-7-azaindole* (**3d**). Conditions: reflux for 3 h. Yield 86%; white solid; mp: 277 °C; ^1^H NMR (200 MHz, DMSO-*d_6_*) δ: 8.54 (dd, *J* = 7.9, 1.2 Hz, 1H, Ar-H), 8.47 (d, *J* = 2.9 Hz, 2H, 2 Ar-H), 8.37 (s, 1H, Ar-H), 8.31 (d, *J* = 1.7 Hz, 1H, Ar-H), 7.64 (d, *J* = 8.8 Hz, 1H, Ar-H), 7.49 (dd, *J* =1.8, 8.7 Hz, 1H, Ar-H), 7.38 (dd, *J* = 4.7, 7.8 Hz, 1H, Ar-H), 3.97 (s, 3H, CH_3_), 3.95 (s, 3H, CH_3_); *Anal.* Calculated for C_19_H_14_BrN_5_O (MW: 408,26): C, 55.90; H, 3.46; N, 17.15%. Found: C, 55.87; H, 3.51; N, 17.27%.

*5-bromo-1-methyl-3-[5-(1-methyl-1H-indol-3-yl)-1,3,4-oxadiazol-2-yl]-7-azaindole* (**3e**). Conditions: 45 °C for 24 h. Yield 78%; white solid; mp:244 °C; ^1^H NMR (200 MHz, DMSO-*d_6_*) δ: 8.62–8.61 (d, *J* = 2.2 Hz, 1H, Ar-H), 8.54–8.53 (d, *J* = 2.3 Hz, 2H, 2 Ar-H), 8.29 (s, 1H, Ar-H), 8.22–8.17 (m, 1H, Ar-H), 7.65–7.61 (dd, *J* = 2.1, 6.1 Hz, 1H, Ar-H), 7.40–7.32 (m, 2H, 2 Ar-H), 3.95 (s, 6H, 2 CH_3_); *Anal.* Calculated for C_19_H_14_BrN_5_O (MW: 408,26): C, 55.90; H, 3.46; N, 17.15%. Found: C, 55.81; H, 3.57; N, 17.23%.

*5-bromo-3-[5-(5-bromo-1-methyl-1H-indol-3-yl)-1,3,4-oxadiazol-2-yl]-1-methyl-7-azaindole* (**3f**). Conditions: reflux for 5 h. Yield 90%; white solid; mp: 275 °C; ^1^H NMR (200 MHz, DMSO-*d_6_*) δ: 8.62 (s, 1H, Ar-H), 8.55 (s, 2H, 2 Ar-H), 8.37 (s, 1H, Ar-H), 8.31 (s, 1H, Ar-H), 7.65 (d, *J* = 8.6 Hz, 1H, Ar-H), 7.50 (d, *J* = 8.6 Hz, 1H, Ar-H), 3.96 (s, 6H, 2CH_3_); *Anal.* Calculated for C_19_H_13_Br_2_N_5_O (MW: 487,16): C, 46.85; H, 2.69; N, 14.38%. Found: 46.93; H, 2.75; N, 14.52%.

*5-bromo-3-[5-(5-fluoro-1-methyl-1H-indol-3-yl)-1,3,4-oxadiazol-2-yl]-1-methyl-7-azaindole* (**3g**). Conditions: r.t. for 24 h. Yield 45%; beige solid mp: 310 °C; ^1^H NMR (200 MHz, DMSO-*d_6_*) δ: 8.69–8.51 (m, 1H, Ar-H); 8.50–8.28 (m, 1H, Ar-H), 8.10 (s, 1H, Ar-H), 7.65 (dd, *J* = 10.0, 2.4 Hz, 2H, 2 Ar-H), 7.56 (dd, *J* = 8.9, 4.4 Hz, 1H, Ar-H), 7.11 (td, *J* = 9.2, 2.3 Hz, 1H, Ar-H), 3.95 (s, 3H, CH_3_), 3.85 (s, 3H, CH_3_); *Anal.* Calculated for C_19_H_13_BrFN_5_O (MW: 426,25): C, 53.54; H, 3.07; N, 16.43%. Found: C, 53.45; H, 3.16; N, 16.51%.

#### 3.1.5. General Procedure for the Synthesis of 3-(5-Phenyl-1,3,4-oxadiazol-2-yl)-7-azaindoles (**4a–d**)

The solution of the proper intermediate **8** (0.81 mmol) in phosphoryl chloride (5.4 mL) was heated under reflux for 3–6 h. Once cooled, the reaction mixture was evaporated in vacuo and extracted with a saturated solution of sodium hydrogen carbonate (3 × 30 mL) and ethyl acetate (3 × 30 mL). The organic layers were evaporated in vacuo and dried with sodium sulfate. The resulting crude material was purified in column using dichloromethane/methanol 99:1 ad eluent.

*3-(5-phenyl-1,3,4-oxadiazol-2-yl)-7-azaindole* (**4a**). Conditions: reflux for 3 h. Yield 70%; white solid; mp272 °C; Spectroscopic data in accordance with [24]. *Anal.* Calculated for C_15_H_10_N_4_O (MW: 262,27): C, 68.69; H, 3.84; N, 21.36%. Found C, 68.78; H, 3.90; N, 21.61%.

*1-methyl-3-(5-phenyl-1,3,4-oxadiazol-2-yl)-7-azaindole* (**4b**). Conditions: reflux for 4 h. Yield 75%; white solid; mp: 201 °C; ^1^H NMR (200 MHz, DMSO-*d_6_*) δ: 8.56 (s, 1H, Ar-H), 8.52 (dd, *J* = 7.9, 1.4 Hz, 1H, Ar-H), 8.47 (d, *J* = 4.7 Hz, 1H, Ar-H), 8.15 (d, *J* = 2.2 Hz, 1H, Ar-H), 8.11 (d, *J* = 3.4 Hz, 1H, Ar-H), 7.67–7.62 (m, 3H, Ar-H), 7.38 (dd, *J* = 4.7, 7.9 Hz, 1H, Ar-H), 3.96 (s, 3H, CH_3_); *Anal.* Calculated for C_16_H_12_N_4_O (MW: 276,30): C, 69.55; H, 4.38; N, 20.28%. Found C, 69.66; H, 4.51; N, 20.41%.

*5-bromo-3-(5-phenyl-1,3,4-oxadiazol-2-yl)-7-azaindole* (**4c**). Conditions: reflux for 3 h. Yield 55%; beige solid; mp: 275 °C; IR (cm^−1^): 3112 (NH); ^1^H NMR (200 MHz, DMSO-*d_6_*) δ: 12.91 (s, 1H, NH), 8.53 (dd, *J* = 10.2, 7.7 Hz, 3H, 3 Ar-H), 8.12 (d, *J* = 3.6 Hz, 2H, 2 Ar-H), 7.64 (d, *J* = 2.2 Hz, 3H, 3 Ar-H); *Anal.* Calculated for C_15_H_9_BrN_4_O (MW: 341,17): C, 52.81; H, 2.66; N, 16.42%. Found C, 52.85; H, 2.60; N, 16.40%.

*5-bromo-1-methyl-3-(5-phenyl-1,3,4-oxadiazol-2-yl)-7-azaindole* (**4d**). Conditions: reflux for 6 h. Yield 60%; white solid; mp: 236 °C; ^1^H NMR (200 MHz, DMSO-*d_6_*) δ: 8.62 (d, *J* = 5.0 Hz, 2H), 8.56 (s, 1H, Ar-H), 8.13 (s, 2H, 2 Ar-H), 7.67 (s, 3H, 3 Ar-H), 3.95 (s, 3H, CH_3_); *Anal.* Calculated for C_16_H_11_BrN_4_O (MW: 355,20): C, 54.10; H, 3.12; N, 15.77%. Found C, 54.10; H, 3.12; N, 15.77%.

#### 3.1.6. General Procedure for the Synthesis of 3-[5-(1-Methyl-1H-indol-3-yl)-1,3,4-thiadiazol-2-yl]-7-azaindoles (**5a–h**)

To a solution of the proper intermediate **7** (0.38 mmol) in pyridine (15 mL), Lawesson’s reagent (0.41 mmol) was added and the reaction mixture was heated under reflux for 4–48 h. The mixture was evaporated in vacuo and the crude material was purified in a chromatography column using dichloromethane/methanol 99:1 as eluent.

*3-[5-(1-methyl-1H-indol-3-yl)-1,3,4-thiadiazol-2-yl]-7-azaindole* (**5a**). Conditions: reflux for 24 h. Yield 78%; beige solid; mp: 246 °C; IR (cm^−1^): 3171 (NH); ^1^H NMR (200 MHz, DMSO-*d_6_*) δ: 12.58 (s, 1H, NH), 8.53 (dd, *J* = 7.9, 1.5 Hz, 1H, Ar-H), 8.42–8.36 (m, 2H, 2 Ar-H), 8.30 (s, 1H, Ar-H), 8.23–8.18 (m, 1H, Ar-H), 7.60 (t, *J* = 9.3 Hz, 1H, Ar-H), 7.39–7.28 (m, 3H, 3 Ar-H), 3.94 (s, 3H, CH_3_); *Anal.* Calculated for C_18_H_13_N_5_S (MW: 331,40): C, 65.24; H, 3.95; N, 21.13%. Found: C, 65.41; H, 3.71; N, 21.32%.

*1-methyl-3-[5-(1-methyl-1H-indol-3-yl)-1,3,4-thiadiazol-2-yl]-7-azaindole* (**5b**). Conditions: reflux for 6 h. Yield 99%; white solid; mp: 244 °C; ^1^H NMR (200 MHz, DMSO-*d_6_*) δ: 8.61 (d, *J* = 7.6 Hz, 1H, Ar-H), 8.44 (d, *J* = 4.5 Hz, 1H, Ar-H), 8.40 (s, 1H, Ar-H), 8.27 (dd, *J* = 6.1, 2.1 Hz, 1H, Ar-H), 8.22 (s, 1H, Ar-H), 7.60 (d, *J* = 6.7 Hz, 1H, Ar-H), 7.41–7.25 (m, 3H, 3 Ar-H), 3.93 (s, 3H, CH_3_), 3.91 (s, 3H, CH_3_); *Anal.* Calculated for C_19_H_15_N_5_S (MW: 345,42): C, 66.07; H, 4.38; N, 20.28%. Found C, 66.23; H, 4.42; N, 20.33; S, 9.35%.

*3-[5-(5-bromo-1-methyl-1H-indol-3-yl)-1,3,4-thiadiazol-2-yl]-1-methyl-7-azaindole* (**5c**). Conditions: reflux for 24 h. Yield 78%; beige solid; mp: 292 °C; ^1^H NMR (200 MHz, DMSO-*d_6_*) δ: 8.56–8.44 (m, 3H, 3 Ar-H), 8.38 (s, 1H, Ar-H), 8.32–8.29 (m, 1H, Ar-H), 7.71–7.63 (m, 1H, Ar-H), 7.52–7.47 (dd, *J* =1.8, 8.8 Hz, 1H, Ar-H), 7.42–7.36 (dd, *J* =4.5, 7.8 Hz, 1H, Ar-H), 3.97 (s, 3H, CH_3_), 3.96 (s, 3H, CH_3_); *Anal.* Calculated for C_19_H_14_BrN_5_S (MW: 424,32): C, 53.78; H, 3.33; N, 16.51%. Found C, 53.81; H, 3.40; N, 16.72%.

*3-[5-(5-fluoro-1-methyl-1H-indol-3-yl)-1,3,4-thiadiazol-2-yl]-1-methyl-7-azaindole* (**5d**). Conditions: reflux for 6 h. Yield 60%; white solid; mp:240 °C; ^1^H NMR (200 MHz, DMSO-*d_6_*) δ: 8.62–8.58 (dd, *J* = 1.5, 7.9 Hz, 1H, Ar-H), 8.46–8.42 (dd, *J* = 1.5, 4.7 Hz, 1H, Ar-H), 8.41 (s, 1H, Ar-H), 8.29 (s, 1H, Ar-H), 8.00–7.94 (dd, *J* = 2.5, 9.8 Hz, 1H, Ar-H), 7.67–7.61 (dd, *J* = 4.5, 8.9 Hz, 1H, Ar-H), 7.39–7.33 (dd, *J* = 4.7, 7.9 Hz, 1H, Ar-H), 7.26–7.16 (td, *J* = 2.5, 9.2, 9.2 Hz, 1H, Ar-H), 3.93 (s, 3H, CH_3_), 3.92 (s, 3H, CH_3_); *Anal.* Calculated for C_19_H_14_FN_5_S (MW: 363,41): C, 62.80; H, 3.88; N, 19.27%. Found: C, 62.89; H, 3.67; N, 19.31%.

*3-[5-(5-methoxy-1-methyl-1H-indol-3-yl)-1,3,4-thiadiazol-2-yl]-1-methyl-7-azaindole* (**5e**). Conditions: reflux for 4 h. Yield 60%; white solid; mp: 200 °C; ^1^H NMR (200 MHz, DMSO-*d_6_*) δ: 8.63–8.37 (m, 3H, 3 Ar-H), 8.21–8.12 (m, 1H, Ar-H), 7.77–7.64 (m, 1H, Ar-H), 7.54–7.47 (dd, *J* = 5.8, 8.9 Hz, 1H, Ar-H), 7.40–7.31 (dt, *J* = 7.9, 3.9 Hz, 1H, Ar-H), 7.00–6.93 (dt, *J* = 2.9, 5.8 Hz, 1H, Ar-H), 3.95 (s, 3H, OCH_3_), 3.90 (s, 3H, CH_3_), 3.87 (s, 3H, CH_3_); *Anal.* Calculated for C_20_H_17_N_5_OS (MW: 375,45): C, 63.98; H, 4.56; N, 18.65%. Found C, 63.98; H, 4.56; N, 18.65%.

*5-bromo-1-methyl-3-[5-(1-methyl-1H-indol-3-yl)-1,3,4-thiadiazol-2-yl]-7-azaindole* (**5f**). Conditions: reflux for 6 h. Yield 70%; beige solid; mp: 233 °C; ^1^H NMR (200 MHz, DMSO-*d_6_*) δ: 8.74–8.73 (d, *J* = 2.2 Hz, 1H, Ar-H), 8.55–8.51 (dd, *J* = 2.2, 5.4 Hz, 1H, Ar-H), 8.47 (s, 1H, Ar-H), 8.30–8.23 (m, 2H, 2 Ar-H), 7.64–7.58 (m, 1H, Ar-H), 7.38–7.29 (m, 2H, 2 Ar-H), 3.95 (s, 3H, CH_3_), 3.91 (s, 3H, CH_3_); *Anal.* Calculated for C_19_H_14_BrN_5_S (MW: 424,32): C, 53.78; H, 3.33; N, 16.51%. Found C, 53.81; H, 3.40; N, 16.62%.

*5-bromo-3-[5-(5-bromo-1-methyl-1H-indol-3-yl)-1,3,4-thiadiazol-2-yl]-1-methyl-7-azaindole* (**5g**). Conditions: reflux for 48 h. Yield 50%; beige solid; mp: 275 °C; ^1^H NMR (200 MHz, DMSO-*d_6_*) δ: 8.60 (d, *J* = 2.1 Hz, 1H, Ar-H), 8.52 (t, *J* = 5.6 Hz, 2H, 2 Ar-H), 8.35 (s, 1H, Ar-H), 8.28 (s, 1H, Ar-H), 7.61 (t, *J* = 7.4 Hz, 1H, Ar-H), 7.52–7.39 (m, 1H, Ar-H), 3.95 (s, 3H, CH_3_), 3.91 (s, 3H, CH_3_); *Anal.* Calculated for C_19_H_13_Br_2_N_5_S (MW: 503,22): C, 45.35; H, 2.60; N, 13.92%. Found C, 45.45; H, 2.65; N, 13.99%.

*5-bromo-3-[5-(5-fluoro-1-methyl-1H-indol-3-yl)-1,3,4-thiadiazol-2-yl]-1-methyl-7-azaindole* (**5h**). Conditions: reflux for 6 h. Yield 80%; white solid; mp: 290 °C; ^1^H NMR (200 MHz, DMSO-*d_6_*) δ: 8.59–8.45 (m, 3H, 3 Ar-H), 8.28 (s, 1H, Ar-H), 7.97–7.81 (qd, *J* = 2.3, 9.5 Hz, 1H, Ar-H), 7.68–7.59 (m, 1H, Ar-H), 7.25–7.16 (m, 1H, Ar-H), 3.94 (s, 3H, CH_3_), 3.90 (s, 3H, CH_3_); *Anal.* Calculated for C_19_H_13_BrFN_5_S (MW: 442,31): C, 51.59; H, 2.96; N, 15.83%. Found C, 51.78; H, 2.85; N, 15.89%.

#### 3.1.7. General Procedure for the Synthesis of 3-[5-(1-Methyl-1H-indol-3-yl)-1,3,4-thiadiazol-2-yl]-7-azaindoles (**6a–d**)

To a solution of the proper intermediate **8** (0.38 mmol) in pyridine (15 mL), Lawesson’s reagent (0.41 mmol) was added, and the reaction mixture was heated under reflux for 24–48 h. The mixture was evaporated in vacuo and the crude material was purified in a chromatography column using dichloromethane/methanol 99:1 as eluent.

*3-(5-phenyl-1,3,4-thiadiazol-2-yl)-7-azaindole* (**6a**). Conditions: reflux for 24 h. Yield: 78%; white solid; mp: 249 °C; IR (cm^−1^): 3111 (NH); ^1^H NMR (200 MHz, DMSO-*d_6_*) δ: 12.56 (s, 1H, NH), 8.61–8.57 (dd, *J* = 1.4, 7.9 Hz, 1H, Ar-H), 8.41 (s, 1H, Ar-H), 8.39–8.38 (d, *J* = 1.4 Hz, 1H, Ar-H), 8.03–7.98 (m, 2H, 2 Ar-H), 7.60 (s, 1H, Ar-H), 7.58–7.57 (d, *J* = 2.7 Hz, 2H, 2 Ar-H), 7.35–7.29 (dd, *J* = 4.7, 7.9 Hz, 1H, Ar-H); *Anal.* Calculated for C_15_H_10_N_4_S (MW: 278,33): C, 64.73; H, 3.62; N, 20.13%. Found C, 64.80; H, 3.91; N, 20.25%.

*1-methyl-3-(5-phenyl-1,3,4-thiadiazol-2-yl)-7-azaindole* (**6b**). Conditions: reflux for 24 h. Yield: 95%; white solid; mp: 191 °C; ^1^H NMR (200 MHz, DMSO-*d_6_*) δ: 8.71–8.70 (d, *J* = 2.2 Hz, 1H, Ar-H); 8.54 (s, 1H, Ar-H), 8.52–8.51 (d, *J* = 2.2 Hz, 1H, Ar-H), 8.06–7.99 (m, 2H, 2 Ar-H), 7.70–7.69 (d, *J* = 2.1 Hz, 1H, Ar-H), 7.60–7.57 (t, *J* = 3.2 Hz, 3H, 3 Ar-H), 3.91 (s, 3H, CH_3_); *Anal.* Calculated for C_16_H_12_N_4_S (MW: 292,36): C, 65.73; H, 4.14; N, 19.16%. Found C, 65.65; H, 4.20; N, 19.31%.

*5-bromo-3-(5-phenyl-1,3,4-thiadiazol-2-yl)-7-azaindole* (**6c**). Conditions: reflux for 24 h. Yield: 60%; white solid; mp: 308 °C; IR (cm^−1^): 3111 (NH); ^1^H NMR (200 MHz, DMSO-*d_6_*) δ: 12.68 (s, 1H, NH), 8.73–8.72 (d, *J* = 2.2 Hz, 1H, Ar-H), 8.49 (s, 1H, Ar-H), 8.47–8.46 (d, *J* = 2.2 Hz, 1H, Ar-H), 8.02–8.01 (d, *J* = 3.5 Hz, 1H, Ar-H), 7.99–7.98 (d, *J* = 2.5 Hz, 1H, Ar-H), 7.60–7.57 (t, *J* = 3.2 Hz, 3H, 3 Ar-H); *Anal.* Calculated for C_15_H_9_BrN_4_S (MW: 357,23): C, 50.43; H, 2.54; N, 15.68%. Found C, 50.49; H, 2.40; N, 15.77%.

*5-bromo-1-methyl-3-(5-phenyl-1,3,4-thiadiazol-2-yl)-7-azaindole* (**6d**). Conditions: reflux for 48 h. Yield: 82%; beige solid; mp: 188 °C; ^1^H NMR (200 MHz, DMSO-*d_6_*) δ: 8.73–8.72 (d, *J* = 2.2 Hz, 1H, Ar-H), 8.57 (s, 1H, Ar-H), 8.54–8.53 (d, *J* = 2.3 Hz, 1H, Ar-H), 8.04–8.03 (d, *J* = 3.3 Hz, 1H, Ar-H), 8.00–7.99 (d, *J* = 2.5 Hz, 1H, Ar-H), 7.61–7.58 (t, *J* = 3.3 Hz, 3H, 3 Ar-H), 3.92 (s, 3H, CH_3_); *Anal.* Calculated for C_16_H_11_BrN_4_S (MW: 371,26): C, 51.76; H, 2.99; N, 15.09%. Found C, 51.70; H, 2.79; N, 15.25%.

### 3.2. Biology

#### 3.2.1. Drugs and Chemicals

Each compound was initially dissolved in dimethyl sulfoxide (DMSO), in order to obtain a 10 mM stock solution, stored at +4 °C, which was then diluted in a complete culture medium immediately before use at the appropriate concentration. The medium, Fetal Bovine Serum (FBS), penicillin and streptomycin were from Gibco (Gaithersburg, MD, USA).

#### 3.2.2. Culture

For the in vitro experiments, we selected seven models of PDAC, including BxPC-3, Panc-1, Suit-2, Capan-1, and PaTu-T cells, the primary culture PDAC-3 and the gemcitabine-resistant cells Panc-1-GR.

BxPC-3 are cells isolated from a pancreas adenocarcinoma, producing mucin, and exhibiting an epithelial phenotype. Of note, BxPC-3 cells lack a KRAS mutation, although this is found in more than 90% of PDAC cases. BxPC-3 cells have a high expression of the angiogenic factors IL-8, VEGF, and PGE2, which can serve as potential additional drug targets. BxPC-3 cells were cultured in RPMI-1640 (Roswell Park Memorial Institute 1640) medium supplemented with 10% NBCS (New Born Calf Serum) and 1% penicillin/streptomycin (Pen/Strep) in T-75 flasks.

Suit-2 is a cell line derived from a metastatic liver tumor of PDAC carcinoma. Suit-2 cell line produces and releases at least two tumor markers, carcinoembryonic antigen and carbohydrate antigen. Under an electron microscope, they appear to be morphologically heterogeneous (i.e., both fusiform and polygonal). Cells were cultured in RPMI-1640 supplemented with 10% heat-inactivated NBCS and 1% Pen/Strep solution in T-75 flasks.

Capan-1 cells are adherent epithelial-like cells derived from a liver metastasis of a PDAC that grew in tissue culture and appeared as large epithelial-like mucin-producing cells. They are characterized by large, relatively dark stained nuclei and abundant slightly basophilic, finely granular, reticulated, or vacuolated cytoplasm. Cells were cultured in Dulbecco’s Modified Eagle’s Medium (DMEM) supplemented with 10% NBCS and 1% Pen/Strep in T-75 flasks.

PaTu-T cells, originating from liver metastases of human pancreatic adenocarcinoma, are highly invasive with significant metastatic potential. PaTu-T has the KRAS G12V mutation and a missense mutation in p53. PaTu-T cells were cultured in DMEM medium supplemented with 10% NBCS and 1% Pen/Strep in T-75 flasks.

Panc-1 is an epithelioid carcinoma-attached cell line, that is commonly used as an in vitro model to study PDAC carcinogenesis and tumor therapies, given the presence of the SSTR2 receptors, prognostic markers in pancreatic cancer. Specifically, these receptors and the presence of neuroendocrine differentiation make this cell line suitable for the neuroendocrine chemotherapy of pancreatic cancer and for radionuclide therapy of the peptide receptor. Panc-1 cells appear to be morphologically heterogeneous (i.e., both cuboid and focally sarcomatoid) and typically display a perinuclear Golgi, scattered or apically localized cytoplasmic vesicles, and many apical plasma membrane microvilli. Panc-1 cells were cultured in RPMI-1640 medium supplemented with 10% NBCS and 1% Pen/Strep in T-75 flasks.

Panc-1-GR (gemcitabine-resistant Panc-1) is a gemcitabine-resistant sub-clone obtained by the continuous exposure of Panc-1 to gemcitabine for 6 days, as previously described [25]. Panc-1-GR were cultured in RPMI medium supplemented with 10% NBCS and 1% Pen/Strep in T-75 flasks.

PDAC-3 is a primary cell line, obtained from a patient undergoing pancreaticoduodenectomy. This cellular model was chosen as previous studies have showed that its genetic and histological characteristics are similar to the original tumor. Cells were cultured in RPMI medium supplemented with 10% NBCS and 1% Pen/Strep in T-75 flasks.

All the cells were maintained under conditions where they are actively growing and undergoing mitotic division, in a humidified incubator at 37 °C with a supply of 5% CO_2_, 95% air atmosphere and 100% relative humidity. Each compound was initially dissolved in dimethyl sulfoxide (DMSO) to obtain a 10 mM stock solution, stored at +4 °C, which was then diluted in a complete culture medium immediately before use at the appropriate concentration.

The cell lines were routinely tested for mycoplasma, while their authentication was performed by short tandem repeat-polymerase chain reaction at BaseClear (Leiden, The Netherlands).

#### 3.2.3. Viability Assay In Vitro

The cytotoxic activity of the nortopsentin derivatives **3b**, **3e**, **4c**, **5b**, and **6c** on pancreatic cancer cells (BxPC-3, Panc-1, Suit-2, Capan-1, PaTu-T, primary PDAC-3 and resistant Panc-1-GR cells) was determined by the sulforhodamine B (SRB) chemosensitivity assay, as described previously [10]. Cells were plated in 96-well flat-bottom plates at final concentrations ranging from 3000–5000 cells/well in 100 µL of medium. After a 24 h pre-incubation period, cells were treated with the compounds at nine screening concentrations (from 0.1 μM to 40 μM) in triplicate and incubated at 37 °C for 72 h. After the treatment, cells were fixed with 25 µL of cold 50% trichloroacetic acid (TCA) for each well and incubated at 4 °C for 1 h. Afterwards, plates were washed five times with demi-water and air-dried overnight. Then, the plates were stained with 50 µL of 0.4% SRB solution in 1% acetic acid for 15 min. The excess stain was rinsed off by placing the plates under running 1% acetic acid and allowed to dry at room temperature overnight. SRB staining was rinsed with 150 µL tris(hydroxymethyl)aminomethane solution pH = 8.8 (TRIS-base), and the optical density (OD) was read at 492 nm. Each assay was performed in triplicate and assays were repeated at least three times. The comparison of the average optical density of the growth in control wells with that in the sample wells allowed the estimation of the percentage of cell growth, using the following equation:

% Cell Growth = (mean ODcompound − mean ODday zero plate)/(mean ODcells − mean ODday zero plate) × 100

The results obtained were adjusted by the day zero plate (wells containing cells growing for only 24 h) and normalized by the control cells (wells with untreated cells) to obtain the rate of viable cells.

#### 3.2.4. Cell-Cycle Analysis

Cell cycle perturbation was analyzed by flow cytometry. Optimized numbers of cells (5 × 10^5^ cells/mL) were seeded in 6-well plates. After an overnight incubation at 37 °C, the cells were treated with the compound **3b**, at two concentrations (2 mM and 4-fold IC_50_) and incubated for 24 h. After treatment, the cells were harvested by trypsinization (250 µL trypsin-EDTA/well), incubated until the cells detached, re-suspended using the same medium used for culture, collected in specific FACS (Fluorescence Activated Cell Sorting) tubes, and washed with 1.5 mL phosphate-buffered saline (PBS). The samples were then centrifuged to form a pellet (5 min at 1500 rpm). These pellets were fixed in 1 mL of ice-cold 70% ethanol, added drop by drop during vortex, and placed in the refrigerator at 4 °C. After overnight incubation, the cells were washed twice with PBS, centrifuged (5 min at 1500 rpm), re-suspended in 50 mL of RNase (100 mg/mL) and incubated for 30 min at 37 °C. Finally, 200 mL of propidium iodide solution (PI, 50 mg/mL) was added, and the cell distribution in the various phases of the cell cycle was analyzed in a FACS Calibur instrument. Data analysis was carried out with FACSdiva software (Becton Dickinson), while cell cycle distribution was determined using Modfit software (Verity-Software, Topsham, ME, USA).

#### 3.2.5. Wound-Healing Assay

Cell migration was assessed using a wound-healing assay, as described previously [26]. A total of 5 × 10^4^ cells/well were seeded in a 96-well plate, to form a confluent monolayer. Scratches were created in confluent cell layers using the sterile scratch tool. The detached cells following scratch induction were removed and new medium was added to the wells. Cells were next treated with compounds **3b** and **5b** at a concentration of 4 × IC_50_. Cells growing in a complete medium were maintained at 37 °C with a supply of 5% CO_2_/95% air atmosphere and 100% relative humidity. The progress of wound closure was observed and documented at various time points (0th, 4th, 8th, 20th, and 24th h) using phase-contrast microscopy. Pictures of the plates were taken using the Universal Grab 6.3 software (DCILabs) from a computer connected to a Leica microscope with a JAI TMC-1327 camera.

The percentage of migration was calculated using the following equation:

%Migration = (Width of the wound at T = 0 − Width of the wound at T = X)/(Width of the wound at T = 0) × 100

#### 3.2.6. Enzyme-Linked Immunosorbent Assay (ELISA) for CDK1 Expression

The expression of CDK1 was detected and quantified using an Enzyme-Linked Immunosorbent Assay (ELISA, Human cyclin-dependent kinase 1 (CDK1) ELISA Kit, Catalog Number: MBS707090) according to the manufacturer’s protocol and our previous studies [27]. Volumes of 100 µL of the standard and the sample were added for each well, and the plates were incubated for 2 h at 37 °C. Then, the liquid was removed, and 100 µL of biotin-antibody was added to each well; the plates were then incubated for 1 h at 37 °C. The medium was removed, and the plates were washed with wash buffer (200 µL). Subsequently, 100 µL of HRP-avidin was added to each well, and the plates were incubated for 1 h at 37 °C. The washing process was repeated five times. At the end, 90 µL of TMB substrate was added to each well, and the plates were incubated for 30 min at 37 °C in the dark. Finally, 50 µL of stop solution was added to each well. The optical density of each well was determined using a microplate reader set to 450 nm.

#### 3.2.7. Statistics

All the SRB and cell-cycle assays were carried out in triplicate and repeated at least three times, whereas the percentages of cell migration were calculated taking into account at least six scratch areas. The data were evaluated using the GraphPad Prism v. 5 software (GraphPad Software, San Diego, CA, USA). Data were expressed as mean values ± SEM and analyzed by the Student *t*-test. *p* values < 0.05 were considered significant (*).

## 4. Conclusions

In the present study, we efficiently synthesized a new series of 1,3,4-oxadiazole and 1,3,4-thiadiazole derivatives **3-6** that showed promising antiproliferative activities in vitro against a panel of PDAC cells, including a primary culture and a resistant variant. Particular efficacy was observed for compound **3b** against all PDAC preclinical models, showing an IC_50_ value of 1.4 μM and the ability to modulate the cell cycle and induce apoptosis. Through wound-healing assays, we also found a remarkable reduction in cell migration in all cell lines, when treated with the most promising compound **3b**. Finally, an ELISA assay in Suit-2 and PDAC-3 cells revealed the significant inhibition of CDK1. As illustrated in Figure 6, this kinase emerges as a potential downstream target of our novel compounds, which might explain all the above-mentioned effects on PDAC cells as well as other potential beneficial anticancer activities demonstrated in previous studies [8]. The overall results of this study warrant further investigation, including both in vivo experiments, with the strongest compounds emerging from in vitro studies, and translational studies for patient therapy stratification based on CDK1 expression. These subsequent studies can provide valuable insights into the potential use of CDK1 expression as a stratification factor for patient therapy, with the aim of improving chemoresistance in PDAC. In conclusion, these findings emphasize the need for the further exploration of CDK1 as a potential therapeutic target for the treatment of PDAC.

## Figures and Tables

**Table 1:** 1,3,4-oxadiazoles **3**,**4** and 1,3,4-thiadiazoles **5**,**6** and their relative yields.

Compound	R	R_1_	R_2_	Yield%	Compound	R	R_1_	R_2_	Yield%
**3a**	H	H	H	75	**5a**	H	H	H	78
**3b**	CH_3_	H	H	86	**5b**	CH_3_	H	H	99
**3c**	CH_3_	H	F	85	**5c**	CH_3_	H	Br	78
**3d**	CH_3_	H	Br	86	**5d**	CH_3_	H	F	60
**3e**	CH_3_	Br	H	78	**5e**	CH_3_	H	OCH_3_	60
**3f**	CH_3_	Br	Br	90	**5f**	CH_3_	Br	H	70
**3g**	CH_3_	Br	F	45	**5g**	CH_3_	Br	Br	50
**4a**	H	H	-	70	**5h**	CH_3_	Br	F	80
**4b**	CH_3_	H	-	75	**6a**	H	H	-	78
**4c**	H	Br	-	55	**6b**	CH_3_	H	-	95
**4d**	CH_3_	Br	-	60	**6c**	H	Br	-	60
					**6d**	CH_3_	Br	-	82

**Table 2:** Cell growth percentages^a^ after 72 h of exposure to 10 μM of the most active compounds **3b**, **3e**, **4c**, **5b**, **6c**.

PDAC Cell Lines	1,3,4-Oxadiazoles			1,3,4-Thiadiazoles	
	3b	3e	4c	5b	6c
BxPC-3	11.10	58.64	21.13	32.88	54.59
Panc-1	9.12	61.96	74.33	15.68	83.68
Panc-1-GR	22.47	>100	98.62	40.65	>100
Suit-2	18.34	54.94	60.18	19.46	29.80
Capan-1	18.33	59.84	75.17	30.86	76.76
PaTu-T	17.13	72.33	31.22	27.73	56.09
PDAC-3	38.35	>100	96.60	55.90	87.84

^a^ Data were expressed as mean values of three replicates.

**Table 3:** Half-maximal inhibitory concentration values (IC_50_) ^a^ in µM of derivatives **3b**, **3e**, **4c**, **5b**, **6c** on pancreatic cancer cells.

PDAC Cell Lines	1,3,4-Oxadiazoles			1,3,4-Thiadiazoles	
	3b	3e	4c	5b	6c
BxPC-3	2.1 ± 0.006	ND ^b^	3.3 ± 2.4	2.7 ± 0.68	ND
Panc-1	2.5 ± 0.55	ND	13.4 ± 3.2	3.5 ± 0.01	11.5 ± 1.2
Panc-1-GR	8.0 ± 2.8	ND	10.8 ± 1.4	9.8 ± 0.05	ND
Suit-2	1.4 ± 0.74	9.4 ± 4.3	ND	1.9 ± 0.27	4.1 ± 0.9
Capan-1	2.6 ± 0.62	ND	10.8 ± 0.67	3.3 ± 0.61	12.6 ± 1.3
PaTu-T	3.2 ± 0.42	ND	9.7 ± 0.17	3.4 ± 0.06	3.8 ± 1.8
PDAC-3	8.1 ± 0.14	23 ± 0.06	ND	10.6 ± 0.04	ND

^a^ SEM: Standard Error of the Mean. ^b^ ND not determined.

## Data Availability

Not applicable.
